# Recently Acquired *Toxoplasma gondii* Infection, Brazil

**DOI:** 10.3201/eid1204.051081

**Published:** 2006-04

**Authors:** Jeffrey L. Jones, Cristina Muccioli, Rubens Belfort, Gary N. Holland, Jacquelin M. Roberts, Claudio Silveira

**Affiliations:** * Centers for Disease Control and Prevention, Atlanta, Georgia, USA;; † Federal University of São Paulo Paulista School of Medicine, São Paulo, São Paulo, Brazil;; ‡ David Geffen School of Medicine at UCLA, Los Angeles, California, USA;; § Clinica Silveira, Erechim, Rio Grande do Sul, Brazil

**Keywords:** Toxoplasmosis, Toxoplasma gondii, epidemiologic study, survey, ocular disease, South America, Brazil

## Abstract

The city of Erechim, Brazil, has a 17% prevalence of ocular toxoplasmosis, and type 1 *Toxoplasma gondii* predominates. To examine risk factors for acute *T. gondii* infection in this area, we administered a questionnaire to recently infected persons (n = 131) and seronegative controls (n = 110). Eating undercooked meat; having a garden; working in the garden or yard more than once per week; eating rare meat; eating cured, dried, or smoked meat; eating frozen lamb; and being male increased risk for *T. gondii* infection in univariate analysis. Risk factors independently associated with acute *T. gondii* infection in multivariate analysis were working in the garden (odds ratio [OR] 2.35, 95% confidence interval [CI] 1.27–4.33) and eating frozen lamb (OR 2.06, 95% CI 1.15–3.67). Among women (n = 86), having had children markedly increased the risk for *T. gondii* infection (OR 14.94, 95% CI 3.68–60.73).

## Abstract 2

Soil exposure, eating undercooked meat, and having children are risk factors for acute infection and high rate of eye disease.

In recent years, because of the large number of postnatal *Toxoplasma gondii* infections, researchers estimate that most ocular disease from *T. gondii* is caused by infection after birth (1–3). In Erechim, a city in southern Brazil, a representative population-based household survey showed that 17.7% of >1,000 persons examined had ocular toxoplasmosis (4). This high rate is believed to be due to acute infection after birth because the rate of infection in young children in this area is low (4–6). Erechim has a population of 96,310 and is located in northern Rio Grande do Sul (population 9,619,416), the southernmost state of Brazil, which borders Uruguay and Argentina. The area was mostly settled by Italian, German, and Polish immigrants in the early 20th century. Of the 3 primary restriction fragment polymorphism (SAG2) genetic types of *T. gondii*, investigators have primarily found the type 1 organism in southern Brazil (7) and have identified it from chickens (*8*,*9*). Type 1 *T. gondii* is uncommon in the United States and may be more virulent for ocular disease (10,11). However, with worldwide food and animal distribution it could become more common in the United States and other countries.

Although ocular toxoplasmosis has been a problem in southern Brazil for many years, no controlled studies have determined the sources of infection. Therefore, in 2003 and 2004, we conducted a case-control study at the principal ophthalmology clinic in Erechim to evaluate the risk factors responsible for *T. gondii* infection so that prevention messages could be tailored to the factors identified.

## Methods

The study was conducted by ophthalmologists at Clinica Silveira and the Federal University at São Paulo, Brazil, in collaboration with researchers from the University of California, Los Angeles, California, and the Centers for Disease Control and Prevention (CDC), Atlanta, Georgia. The questionnaire was adapted from those used in previous *T. gondii* case-control studies (12,13), with modification and input from the study researchers. The questionnaire took ≈25 minutes to complete and inquired about demographic variables and a comprehensive set of risk factors related to meat, vegetables, food preparation, soil contact, drinking water, and animal exposure (especially cat and cat feces exposure). Information about the number of pregnancies and children was collected from women participating in the survey. Questions involving habitual behavior focused on the most recent 12 months. The questionnaire was self-administered at Clinica Silveira, the principal ophthalmology clinic in Erechim, with parental assistance for children <16 years of age.

The study was reviewed and approved by the human subjects review committee at the Federal University of São Paulo and approved for analysis at CDC. Oral consent was obtained for completion of the questionnaire. All participants with ocular disease received care and follow-up at Clinica Silveira.

Case-patients with recent *T. gondii* infection were defined as consecutive visitors to the clinic from June 2003 to June 2004 with positive test results for both *T. gondii*–specific immunoglobulin G (IgG) and IgM. For each patient, an attempt was made to identify a control as the next patient with negative *T. gondii* antibody test results. Antibody testing was performed at the Fleury Laboratory by using the Abbott AxSYM system (Abbott Diagnostics, Abbott Park, IL, USA).

Data were first examined with univariate analysis; then factors with p value <0.05 for association with recent *T. gondii* infection (and sufficient sample size) were entered into logistic regression models for the sample as a whole (N = 241), for women >18 years of age (n = 86), and children <18 years of age (n = 106). Men >18 years of age were not examined as a subgroup in logistic models because the sample size was too small (n = 49). In odds ratio (OR) calculations with a zero value in >1 cells, a correction of 0.5 was added to every cell to avoid undefined results (logit method). Attributable risks were calculated from the logistic regression results for factors that significantly (p<0.05) increased risk for *T. gondii* infection by using the following formula: attributable risk = proportion of cases exposed for each factor × ([OR – 1]/OR). Data analysis was conducted with SAS software (14).

## Results

Of 140 persons who had positive *Toxoplasma* IgG and IgM test results, 131 (93.6%) agreed to participate and completed questionnaires. Of 121 persons who had negative *Toxoplasma* IgG and IgM test results, 110 (90.9%) agreed to participate and completed questionnaires. The demographics of patients and controls are presented in Table 1. Controls were more likely to be in the 16- to 20- and 21- to 30-year age ranges and female and less likely to be in the <5-, 31–40-, and >41-year age groups.

**Table 1:** Demographics of *Toxoplasma gondii* patients and controls, Erechim, Brazil, 2003–2004, N=241

Factor	Patients, no. (%) (n = 131*)	Controls, no. (%) (n = 110*)	χ^2^ p value
Age (y)			<0.0001
<5	14 (10.7)	1 (0.9)	
6–10	16 (12.2)	16 (14.6)	
11–15	19 (14.5)	14 (12.7)	
16–20	12 (9.2)	31 (28.2)	
21–30	21 (16.0)	31 (28.2)	
31–40	28 (21.4)	11 (10.0)	
>41	21 (16.0)	6 (5.5)	
Sex			0.01
Male	72 (55.0)	42 (38.2)	
Female	59 (45.0)	68 (61.8)	
Race			0.54
White	127 (97.0)	105 (95.5)	
Other	4 (3.1)	5 (4.5)	
Ethnicity			0.51
German, Polish, or Italian	117 (94.4)	102 (96.2)	
Other	7 (5.7)	4 (3.8)	

*Numbers within each category may not add to total n because of nonresponse.

In univariate analysis among the group as a whole, patients were significantly more likely than controls to eat rare meat (this question referred to meat in general); eat cured, dried, or smoked meat given by a friend or relative (purchased cured, dried, or smoked meat was not significantly associated with case status); have a garden; work in the yard or garden; eat frozen lamb; and be male. Patients were less likely to eat raw beef, eat raw ground beef, eat raw ground chicken, and be born in Erechim or upper Uruguay (Table 2). Among women >18 years of age, having had >1 child (OR 14.68, 95% confidence interval [CI] 4.10–52.50, p<0.0001) (or >1 pregnancy, colinear with number of children) was strongly associated with risk for *T. gondii* infection (Table 3). Women infected with *T. gondii* were also more likely than those not infected to have lived in their present home >12 months, work with animals (all types), work in the garden, and have >1 cat or kitten living at home. They were less likely to be infected if they ate raw ground beef, raw chicken, rare or raw ground chicken, and fed their cat dried food. In addition, the trend in risk for recent *T. gondii* infection increased from having no children (4 [17.4%] of 23), to 1 child (11 [68.8%] of 16), to >2 children (23 [79.3%] of 39) (p<0.0001).

**Table 2:** Factors associated with risk for acute *Toxoplasma gondii* infection, Erechim, Brazil, 2003–2004, N=241 (univariate factors shown with p<0.05)*

Factor	No. with factor/no. patients† (%)	No. with factor/no. controls† (%)	OR (95% CI)	p value
Have a garden (excludes those living on farms)	59/69 (85.5)	41/61 (67.2)	2.88 (1.22–6.78)	0.01
Born in Erechim or upper Uruguay	78/130 (60.0)	98/110 (89.1)	0.18 (0.09–0.37)	<0.0001
Work in garden	57/130 (43.9)	30/110 (27.3)	2.08 (1.21–3.59)	0.008
Eat cured, dried, or smoked meat given by friend or relative	60/130 (46.2)	33/110 (30.0)	2.00 (1.17–3.41)	0.01
Eat rare meat	73/131 (55.7)	47/110 (42.7)	1.69 (1.01–2.81)	0.045
Of those that eat raw meat, eat raw beef	18/31 (58.1)	14/16 (87.5)	0.20 (0.04–1.02)	0.042‡
Of those that eat raw meat, eat raw ground beef	11/31 (35.5)	12/16 (75.0)	0.18 (0.05–0.71)	0.01
Of those that eat raw meat, eat raw ground chicken	2/30 (6.7)	5/15 (33.3)	0.14 (0.02–0.86)	0.02
Eat frozen lamb	37/131 (28.2)	19/110 (17.3)	1.89 (1.01–3.52)	0.045
Male sex	72/131 (55.0)	42/110 (38.2)	1.98 (1.18–3.31)	0.01
Work in yard >1×/wk	65/90 (72.2)	35/71 (49.3)	2.67 (1.39–5.15)	0.003

*OR, odds ratio; CI, confidence interval. †Totals vary because some questions applied to a subset of participants and because response rates varied. ‡Exact method, OR 0.20, 95% CI 0.02–1.14, p = 0.078.

**Table 3:** Factors associated with risk for acute *Toxoplasma gondii* infection in women >18 years of age, Erechim, Brazil, 2003–2004, N=86 (univariate factors shown with p<0.05)*

Factor	No. with factor/no. patients† (%)	No. with factor/no. controls† (%)	OR (95% CI)	p value
Lived in present home >12 mo	46/46 (100)	35/40 (87.5)	14.41 (logit) (0.77–269.25)	0.01
Work with animals	16/46 (34.8)	6/40 (15.0)	3.02 (1.05–8.71)	0.04
Work in garden in past 12 mo	26/46 (56.5)	14/40 (35.0)	2.41 (1.01–5.78)	0.05
Eat rare ground chicken	1/23 (4.4)	4/15 (26.7)	0.13 (0.01–1.26)	0.05
Eat raw ground beef	4/12 (33.3)	6/7 (85.7)	0.08 (0.01–0.95)	0.03
Eat raw chicken	2/12 (16.7)	4/6 (66.7)	0.10 (0.01–0.98)	0.04
Eat raw ground chicken	0/12	4/6 (66.7)	0.02 (logit) (0.00–0.58)	0.001
>1 cat living at home	20/46 (43.5)	9/40 (22.5)	2.65 (1.03–6.81)	0.04
>1 kitten living at home	9/46 (19.6)	2/39 (5.1)	4.50 (0.91–22.26)	0.05
Feed cat dried food	29/46 (63.0)	33/40 (82.5)	0.36 (0.13–0.995)	0.05
>1 pregnancy	34/37 (91.9)	10/28 (35.7)	20.40 (4.98–83.64)	<0.0001
>1 child	34/38 (89.5)	11/30 (36.7)	14.68 (4.10–52.50)	<0.0001

*OR, odds ratio; CI, confidence interval. †Totals vary because some questions applied to a subset of participants and because response rates varied.

Among children <18 years of age, in univariate analysis those with recent *T. gondii* infection were more likely than those without infection to have a cat that catches its own food; feed the cat raw food; work in the yard more than once per week; eat cured, dried, or smoked meat given by a friend or relative; eat frozen lamb; eat rare meat; eat rare pork; and be male. They were less likely to be born in Erechim or upper Uruguay or wear gloves while working in the yard (Table 4).

**Table 4:** Factors associated with risk for acute *Toxoplasma gondii* infection among children <18 years of age, Erechim, Brazil, 2003–2004, N=106 (univariate factors with p<0.05)*

Factor	No. with factor/no. patients† (%)	No. with factor/no. controls† (%)	OR (95% CI)	p value
Born in Erechim or upper Uruguay	32/58 (55.2)	46/48 (95.8)	0.05 (0.01–0.24)	<0.0001
Cat at least occasionally catches own food	25/26 (96.2)	16/21 (76.2)	7.81 (0.83–73.15)	0.04
Feed cat raw food	15/26 (57.7)	6/21 (28.6)	3.41 (1.00–11.61)	0.04
Work in yard >1×/wk	35/43 (81.4)	18/32 (56.3)	3.40 (1.21–9.61)	0.02
Wear gloves when working in yard	0/49	6/35 (17.1)	0.05 (logit) (0.00–0.84)	0.003
Eat cured, dried, or smoked meat given by friend or relative	20/58 (34.5)	7/48 (14.6)	3.08 (1.17–8.11)	0.02
Eat frozen lamb	12/58 (20.7)	3/48 (6.3)	3.91 (1.03–14.80)	0.03
Eat rare meat	33/58 (56.9)	17/48 (35.4)	2.41 (1.10–5.29)	0.03
Of those that eat rare meat, eat rare pork	10/32 (31.3)	0/17	16.33 (logit) (0.89–298.33)	0.01
Male sex	45/58 (77.6)	20/48 (41.7)	4.84 (2.09–11.26)	0.0002

*OR, odds ratio; CI, confidence interval. †Totals vary because some questions applied to a subset of participants and because response rates varied.

In multivariate analysis for the complete group, persons recently infected with *T. gondii* were significantly more likely than those who were not infected to work in the garden (OR 2.35, 95% CI 1.27–4.33, p = 0.006) and eat frozen lamb (OR 2.06, 95% CI 1.15–3.67, p = 0.02) but less likely to be born in Erechim or upper Uruguay (OR 0.19, 95% CI 0.09–0.39, p<0.0001) (Table 5). In multivariate analysis, for women >18 years of age, having had >1 child compared to having no children markedly increased risk for recent *T. gondii* infection (OR 14.94, 95% CI 3.68–60.73, p<0.0002) (Table 5). For children <18 years of age, being born in Erechim or upper Uruguay was associated with a reduced risk for recent *T. gondii* infection compared to those born elsewhere (OR 0.04, 95% CI 0.01–0.22, p = 0.0002), and male sex (OR 5.70, 95% CI 1.95–16.66, p = 0.002) was associated with an increased risk for *T. gondii* infection (Table 5).

**Table 5:** Factors associated with risk for acute *Toxoplasma gondii* infection in multivariate analysis, Erechim, Brazil, 2003–2004

Factor	OR (95% CI)*	p value
All persons (N = 241), factors representing >100 cases and p<0.05 in univariate analysis		
	Born in Erechim or upper Uruguay	0.19 (0.09–0.39)	<0.0001
	Work in garden	2.35 (1.27–4.33)	0.006
	Eat cured, dried, or smoked meat given by friend or relative	1.58 (0.86–2.91)	0.14
	Eat rare meat	1.35 (0.76–2.41)	0.31
	Eat frozen lamb	2.06 (1.15–3.67)	0.02
	Male sex	1.21 (0.59–2.49)	0.60
Women >18 y (n = 86), factors representing >30 cases and p<0.05 in univariate analysis†		
	>1 child	14.94 (3.68–60.73)	0.0002
	Work with animals	1.74 (0.24–12.86)	0.59
	>1 cat living at home	0.63 (0.05–7.35)	0.71
	>1 kitten living at home	2.95 (0.21–41.03)	0.42
	Feed cat dried food	0.86 (0.04–19.10)	0.93
	Work in the garden in past 12 mo	1.09 (0.29–4.05)	0.90
Children <18 y (n = 106), factors representing >50 cases and p<0.05 in univariate analysis		
	Born in Erechim or upper Uruguay	0.04 (0.01–0.22)	0.0002
	Eat cured, dried, or smoked meat given by a friend or relative	1.75 (0.51–6.02)	0.37
	Eat frozen lamb	4.52 (0.85–23.97)	0.08
	Eat rare meat	2.52 (0.93–6.81)	0.07
	Male sex	5.70 (1.95–16.66)	0.002

*Referent is absence of factor. OR, odds ratio; CI, confidence interval. †The factor "lived in present home >12 mo" had a p value of 0.01 in univariate analysis, but 95% CI calculated with logit crossed 1; therefore, it was not included.

The attributable risks for the factors that significantly increased the risk for recent *T. gondii* infection in logistic regression for the group as a whole (from Table 5) were working in the garden (0.25) and eating frozen lamb (0.15). Among women >18 years of age, having had >1 child was associated with a high attributable risk (0.84). Among children <18 years of age, male sex was associated with an attributable risk of 0.64.

## Discussion

In the study group overall (Table 2), we found that a number of meat- and soil-related factors were associated with recent *T. gondii* infection, which included eating cured, dried, or smoked meat given by a friend or relative; eating rare meat; eating frozen lamb; and having a garden and working in the yard or garden (soil contact). The presence of meat-related factors emphasizes the importance of cooking meat well (>67°C) and not assuming that all home freezing methods will kill *T. gondii* cysts. Having a garden or working in the yard increases the chances of oocyst exposure through soil contact. Men were at increased risk for recent *T. gondii* infection, which could also be related to increased soil contact. To minimize *T. gondii* exposure, gloves should be worn while gardening, and hands should be washed thoroughly afterward with soap and water. Although sample size limitations precluded us from including all the variables in multivariate analysis, working in the garden remained significant in multivariate analysis.

Although the number of persons who ate raw meat was small, eating raw ground chicken was associated with a decreased risk for *T. gondii* infection. Chickens grown commercially are probably not involved in the transmission of *T. gondii* because modern methods reduce soil exposure, chickens are usually frozen for storage before purchase, and they are thoroughly cooked (15). Eating chicken could be associated with a decrease in risk because those who eat chicken may be less likely to eat other meats that are associated with a greater risk. However, free-range chickens in Brazil can be infected with *T. gondii* (9), and eating fresh, undercooked meat from free-range chickens could pose a risk for infection. In addition, eating raw beef or raw ground beef decreased the risk for *T. gondii* infection in univariate analysis, although the sample size was small for this variable as well. Beef is rarely contaminated with *T. gondii* and is not believed to be a consequential source of infection (15), but the role of beef in transmission to humans has not been completely determined. Eating raw ground beef that is contaminated with pork because the grinding machine was not cleaned after grinding pork could increase the risk for *T. gondii* infection. Eating rare meat (all types combined) was a risk factor for *T. gondii* infection. Pork and lamb are the most likely meats to be contaminated with *T. gondii* (15). Although these data are not specifically from Erechim, researchers in southern Brazil have recently reported that 8.7% of 149 fresh pork sausage samples were positive for *T. gondii* by bioassay in mice in the state of Parana (16), and 17% of 286 finishing pigs tested positive for *T. gondii* antibodies with the modified agglutination test in the state of São Paulo (17). Dubey et al. determined that tissue cysts survive in pork for <3 minutes if heated to 64°C (18), so cooking meat (especially pork or lamb) below this temperature could lead to infection.

The strongest factor in analysis of the group overall was the reduced risk for *T. gondii* infection among persons born in Erechim or upper Uruguay compared to persons born elsewhere. This reduction in risk was also significant in multivariate analysis and in children and is likely due to referral of patients with ocular disease from outside the area. Risk for *T. gondii* infection was also reduced among women born in Erechim or upper Uruguay, but the reduction was not significant (data not shown).

For women >18 years of age, having had >1 child was strongly associated with recent *T. gondii* infection in both univariate and multivariate analysis (Tables 3 and 5). Having had >1 pregnancy also showed a strong association with recent *T. gondii* infection in univariate analysis, but we did not examine it in multivariate analysis because it is highly correlated with the number of children. Pregnancy and number of children were risk factors for *T. gondii* infection in other studies, including several from Brazil (19–22). Avelino et al. (20) suggest that the greater vulnerability of pregnant women to *T. gondii* infection is probably due to alterations in immune mechanisms associated with gestation. In some settings, pregnancy could affect culinary habits. In our study, the number of children and past pregnancies increased risk for recent *T. gondii* infection. Another possible explanation for this finding is that children bring, or track, contaminated soil into the house, increasing risk for infection.

Among children <18 years of age, in univariate analysis many of the meat- and soil-related risk factors were associated with an increased risk for infection that was seen in the group as a whole (Table 4). In addition, feeding cats raw food and having cats that catch their own food were associated with increased risk. To prevent cats from becoming infected with *T. gondii*, they should be fed only dry, canned, or well-cooked food and kept indoors when possible to discourage hunting. In multivariate analysis of the risk factors for children, male sex was the principal factor that significantly increased risk for infection, although eating rare meat and frozen lamb approached significance.

One of the strengths of our study is that serologically defined acute *T. gondii* infection was required for the case definition. Most *T. gondii* IgM- and IgG-positive persons are infected for <1 year, so their more recent behavior correlates with the time of their infection. Our study also has a number of limitations. The Clinica Silveira is known for expertise in ocular toxoplasmosis and draws patients with toxoplasmosis-related eye disease from the surrounding area. This fact is the likely reason why patients with acute *T. gondii* infection were more likely to come from outside the Erechim area than from Erechim. Although the sample size was adequate to identify numerous risk factors for *T. gondii* infection, it was not large enough to allow analysis for all subgroups (for example, multivariate analysis of adult men). Some associations in our study may have been due to confounders that we did not consider in the analysis (especially in univariate analysis); however, we inquired about a comprehensive set of variables, including those previously known to be associated with *T. gondii* infection, and performed multivariate analysis. In addition, our study was limited to 1 area of Brazil, and the results cannot necessarily be applied to other areas of the country.

In conclusion, our study identified a number of risk factors for *T. gondii* infection among persons with ocular disease. Because *T. gondii* infection often leads to ocular disease in this region of Brazil, avoiding infection can improve ocular health. Proper meat and soil- and water-related hygiene could reduce the risk for infection with *T. gondii*, especially with preventive education efforts based on risk factors identified in this case-control study. The association of *T. gondii* infection in women with the number of children they have had (and their pregnancies) requires further study to fully elucidate the factors that contribute to this increase in risk. In the future, type 1 *T. gondii* could spread to other areas of the world and increase the risk for ocular disease in those regions.
